# Effect of Full-mouth Disinfection Protocol on Glycaemic Control and Subgingival Microbiota in Patients with Type 1 and Type 2 Diabetes

**DOI:** 10.3290/j.ohpd.b965731

**Published:** 2021-02-19

**Authors:** Nina Hropot Plesko, Eva Skaleric, Katja Seme, Andrej Janez, Uros Skaleric, Boris Gaspirc

**Affiliations:** a Teaching Assistant, Department of Oral Medicine and Periodontology, University of Ljubljana, Faculty of Medicine, Ljubljana, Slovenia. Performed the clinical examinations, wrote the manuscript.; b Teaching Assistant, Department of Oral Medicine and Periodontology, University Medical Centre Ljubljana and Medical Faculty of Ljubljana, Ljubljana, Slovenia. Proofread the manuscript, contributed to discussion.; c Professor, Institute of Microbiology and Immunology, University of Ljubljana, Faculty of Medicine, Ljubljana, Slovenia. Proofread the manuscript, performed the microbiological analysis.; d Professor, Department of Endocrinology, Diabetes and Metabolic Diseases, Division of Internal Medicine, University Medical Centre Ljubljana, Ljubljana, Slovenia. Proofread the manuscript, contributed to the selection of patients.; e Academic Professor, Department of Oral Medicine and Periodontology, University Medical Centre Ljubljana, Ljubljana, Slovenia. Idea, proofread the manuscript, contributed to discussion.; f Assistant Professor, Department of Oral Medicine and Periodontology, University Medical Centre Ljubljana and Medical Faculty of Ljubljana, Ljubljana, Slovenia. Performed the statistical evaluation, proofread the manuscript, contributed to discussion.

**Keywords:** diabetes mellitus, full-mouth disinfection, HbA1c, periodontitis, subgingival microbiota

## Abstract

**Purpose:**

To evaluate the effect of a full-mouth disinfection protocol (FMD) on periodontal parameters, glycaemic control and subgingival microbiota of periodontal patients with type 1 and type 2 diabetes, as well as those without diabetes.

**Materials and Methods:**

This study included 33 patients with periodontitis. Eleven of them were type 1 diabetes patients, 11 were type 2 diabetes patients, and 11 were non-diabetics. At baseline and 3 months after the FMD, the periodontal parameters of each patient were recorded, samples of capillary blood for the chairside assessment of HbA1c were taken, and plaque samples from the two deepest periodontal pockets were collected to test for the presence of *Aggregatibacter actinomycetemcomitans* (Aa), *Porphyromonas gingivalis* (Pg), *Prevotella intermedia* (Pi), *Tannerella forsythia* (Tf) and *Treponema denticola* (Td).

**Results:**

Bleeding on probing (BOP), probing pocket depth (PPD), clinical attachment level (CAL) and glycated haemoglobin (HbA1c) decreased statistically significantly (p < 0.05) in all three groups 3 months after FMD. Only the proportion of Pg in the control group decreased statistically significantly (p < 0.05), while the proportion of other bacteria decreased or remained the same, whereby the differences were not statistically significant. Moreover, the proportion of Aa in type 1 diabetics increased statistically significantly (p < 0.05).

**Conclusion:**

The FMD protocol improves periodontal parameters and glycaemic control of type 1 and type 2 diabetes patients with periodontitis.

Periodontal disease is one of the most common infections in the adult population. It is a chronic inflammatory disease caused by bacteria accumulating and reproducing on the surface of the teeth. It results in the destruction of the supporting structures of the teeth.^19^

Diabetes mellitus is a chronic disease that represents one of the greatest global medical concerns; its prevalence is increasing. The majority of the cases are type 2 diabetes mellitus, which is associated with obesity and mostly occurs in the adult population.^33^ It is well known that diabetes increases the risk and severity of periodontal disease^9,16,17^ and that periodontal disease is associated with glycaemic control of diabetes patients.^12,17,33^

A meta-analysis regarding the effect of periodontal treatment on the glycaemic control of diabetic patients suggests that periodontal treatment improves the glycaemic control in type-2 diabetes patients for a minimum of three months.^30^ Another systematic review examining the effect of periodontal therapy on glycaemic control in diabetics revealed a significant but limited effect of periodontal therapy on improvement of HbA1c in diabetics.^11^ On the other hand, Simpson et al^25^ concluded that therapy of periodontal disease by scaling and root planing improves the level of HbA1c in type 1 and type 2 diabetes patients at 3–4 months, but there is not enough evidence to show that this effect persists after 4 months.

Periodontal treatment improves the glycaemic control of type 1 and type 2 diabetes mellitus patients for at least 3 months.^23,28^ Dag et al,^6^ on the other hand, concluded that non-surgical treatment of periodontal disease in uncontrolled diabetic patients does not significantly improve the level of glycaemia, unless strict glycaemic control is achieved.

Little is known about whether there is a difference in the subgingival microbial flora between diabetics and non-diabetics.^17^ Several studies investigating the microbiota in diabetics compared to non-diabetics have been carried out and found an increased amount of periodontal pathogens in diseased sites of diabetes patients, but specific differences regarding the bacterial species were not found.^15,17,22,35^

Traditionally, non-surgical treatment of periodontal disease is carried out by jaw quadrant or sextant at a series of appointments. During that time, reinfection from untreated periodontal pockets, tongue and tonsils could occur. In contrast, in full-mouth disinfection (FMD) protocol, the root instrumentation of all teeth is completed within 24 h and includes tongue cleaning and extensive use of chlorhexidine.^20,32^

Only a few studies have investigated the influence of the FMD protocol on HbA1c and subgingival microbiota in diabetes patients.^3,21,23,29^ Most of these studies investigated the effect of FMD in type 2 diabetes patients; to the best of our knowledge, only one of the studies investigated the effect of FMD in type 1 diabetes patients.^23^

The aim of our study was to evaluate whether non-surgical therapy with the full-mouth disinfection protocol improves periodontal parameters, leads to a decrease in HbA1c levels, and decreases the proportion of periodontopathogenic bacteria in periodontal pockets of type 1 and 2 diabetes patients as well as non-diabetic patients.

## Subjects and Methods

The study was conducted in accordance with the ethical principles of the Helsinki Declaration (WMA, 2013) and written informed consent was obtained. The study protocol was approved by the National Medical Ethics Committee of the Republic of Slovenia (No. 49/08/11).

### Inclusion Criteria

Patients were recruited from the Department of Endocrinology, Diabetes and Metabolic Diseases and the Department of Oral Diseases and Periodontology at the University Medical Centre Ljubljana. All patients were diagnosed with at least stage II periodontitis, grades B or C (chronic periodontitis). The study encompassed 33 patients: 19 females and 14 males. Eleven of them were diagnosed with periodontitis and type 1 diabetes mellitus, 11 were diagnosed with periodontitis and type 2 diabetes mellitus, and 11 were diagnosed with periodontitis but did not have diabetes mellitus.

### Exclusion Criteria

Subjects were excluded on the basis of the following criteria: 1. use of systemic antibiotic therapy 3 months prior to the baseline examination; 2. periodontal therapy 6 months before the start of the study; 3. pregnancy and breastfeeding; 4. conditions/diseases that shorten the lifespan of red blood cells, including haemolytic anaemia, recent massive blood loss and positive rheumatoid factors (the chairside test for the HbA1c assessment is not valid in these cases); 5. smokers (5 cigarettes or more per day).

We obtained the information from patients through an interview and a questionnaire.

### Study Design

A clinical periodontal examination was performed, recording the number of teeth, probing pocket depth (PPD), gingival recession, clinical attachment level (CAL), bleeding on probing (BOP) and full mouth plaque score (FMPS) at 6 sites around each tooth (disto-buccal, buccal, mesio-buccal, disto-lingual, lingual and mesio-lingual) using a Williams periodontal probe (Hu Friedy; Chicago, IL, USA).

Samples of capillary blood were taken from a fingertip and the HbA1c value was assessed chairside (Affinion Axis-Shield device; Oslo, Norway).^34^ The primary clinical outcome variable of the present study was the HbA1c value, the secondary clinical outcome variables were PPD and BOP values.

Samples of subgingival plaque were obtained with paper points from the two deepest periodontal pockets in each patient.^13^ Each pocket was sampled by one sterile paper point, which was inserted to the depth of the pocket and kept in place for 10-20 s.^18,24^ In total, 132 samples were obtained (66 before and 66 after FMD). Each sampling site was tested for the presence of *Aggregatibacter actinomycetemcomitans* (Aa),* Porphyromonas gingivalis* (Pg), *Prevotella intermedia* (Pi), *Tannerella forsythia* (Tf) and *Treponema denticola* (Td) by a multiplex polymerase chain reaction (PCR) followed by hybridisation against species-specific DNA probes using a commercially available micro-IDent test (Hain Lifescience; Nehren, Germany).^7^ The analysis was performed at the Institute of Microbiology and Immunology, Faculty of Medicine, at the University of Ljubljana.

After the initial periodontal examination, all patients received non-surgical periodontal therapy using the full-mouth disinfection protocol.^20,23^ Supragingival plaque removal and scaling and root planing of all teeth were performed in one session. The tongue was scraped, the teeth were polished and the periodontal pockets were rinsed with a 0.12% chlorhexidine solution. The patients rinsed their mouths for 2 min with a 0.12% chlorhexidine solution. All patients received instructions for proper self-performed oral hygiene, which included rinsing the mouth twice a day for 2 min with a 0.12% chlorhexidine solution for a period of two weeks. After the treatment was performed, patients did not receive any periodontal interventions for three months.

After 3 months, the patients were re-examined and PPD, gingival recession, CAL, BOP and FMPS at 6 sites around each tooth were measured. Samples from the two deepest pockets were taken for a microbiological assessment and HbA1c was measured in the samples of capillary blood.

### Statistical Analysis

To detect a difference of 0.1 mm in PPD between the three treatment groups (α = 0.05, β = 0.20, estimated SD = 1 mm), it was estimated that 10 patients would be required. According to calculation, 1571 measuring sites in each group were needed.

Clinical parameters at baseline and 3 months after the FMD treatment were compared between and within three groups. The primary outcome variable was HbA1c, the secondary outcome variables were PPD and BOP. In addition, microbiological parameters of subgingival plaque samples before treatment and at 3 months were also analysed and compared to baseline and between the groups.

The Friedman test, as a nonparametric analogue for repeated measures ANOVA, and the nonparametric Kruskal-Wallis test were used to compare the BOP, PPD, gingival recession, CAL and HbA1c values between the three groups at baseline and three months after FMD. The Friedman test was also used to compare the proportions of examined periodontal pathogenic bacteria between the three treatment groups at baseline and three months after the FMD treatment. The level of significance was set at α = 0.05, and the statistical power was 0.80.

## Results

Thirty-three patients were included in the study. The characteristics of the population are presented in Table 1. Among diabetes patients, 18.2% were diagnosed with periodontitis stage II, grades B or C, 63.6% were diagnosed with periodontitis stage III, grades B or C, and 18.2% were diagnosed with periodontitis stage IV, grades B or C. In the control group, 27.3% of patients were diagnosed with stage II periodontitis, grades B or C, and 72.7% were diagnosed with stage III periodontitis, grades B or C. According to the former classification of periodontal disease, all patients were diagnosed with chronic periodontitis.

**Table 1:** Characteristics of the study population at baseline

	N	Age (years)	Gender	Duration of diabetes mellitus (years)
Type 1 diabetes mellitus	11	40.3 ± 4.9	F 7, M 4	24.3 ± 9.6
Type 2 diabetes mellitus	11	62.4 ± 5.4	F 6, M 5	15.3 ± 7.3
Control group	11	51.2 ± 4.7	F 6, M 5	

Mean ± standard deviation. N: number of subjects; M: male; F: female; control group: patients with periodontitis but without diabetes mellitus.

### Clinical Outcomes

BOP decreased statistically significantly in all three groups (p < 0.05) 3 months after full-mouth disinfection was performed (Table 2). FMPS decreased in all three treatment groups, with this decrease being statistically significant in the control group and in the type 2 diabetes group (p < 0.05), whereas in the type 1 diabetes group, the decrease was not significant. In the same time period, PPD decreased significantly in all three groups (p < 0.05). Gingival recessions almost did not change after FMD treatment, but a significantly lower recession (p < 0.05) was recorded in the type 1 diabetes mellitus group. CAL decreased in all three treatment groups, with the decrease being statistically significant in the control group (p < 0.05) and not significant in both diabetes groups. The distribution of the shallow (0–4 mm), medium (5–6 mm) and deep pockets (>6 mm) at baseline and 3 months after treatment was evaluated (Table 3). The proportion of shallow pockets increased in all three groups, while the proportion of medium and deep pockets decreased in all three groups.

**Table 2:** Bleeding on probing score, full mouth plaque score, probing depth, gingival recession and CAL in control, type 1 and type 2 diabetes groups of the study population

	Time	Group					
		CG (N=1740)		DM 1 (N=1794)		DM 2 (N=1464)	
BOP (%)	Baseline	57.52	± 4.94	53.30	± 4.99	39.80	± 4.89
	3 months	34.81	± 4.76*	45.53	± 4.98*	35.22	± 4.77*
FMPS (%)	Baseline	52.70	± 13.06	54.34	± 15.80	45.64	± 20.80
	3 months	33.27	± 9.86£	48.91	± 12.86	34.16	±16.63¥
PPD (mm)	Baseline	3.18	± 1.39	3.02	± 1.08	3.05	± 1.23
	3 months	2.75	± 1.08*	2.94	± 0.96*	2.79	± 1.09*
Recession (mm)	Baseline	0.25	± 0.71	0.10	± 0.43	0.35	± 0.90
	3 months	0.24	± 0.72	0.08	± 0.41 *	0.37	± 0.93§
CAL (mm)	Baseline	3.42	± 1.55	3.11	± 1.17	3.40	± 1.45
	3 months	3.00	± 1.28*	3.02	± 1.06	3.16	± 1.38

Data are given as means ± SD, N=number of measured sites. DM 1: type 1 diabetes mellitus; DM 2: type 2 diabetes mellitus; CG: control group. *p < 0.05 vs baseline, §p < 0.05 vs DM 1, £p < 0.05 vs baseline, ¥p < 0.05 vs baseline.

**Table 3:** Distribution of shallow (0-4 mm), medium (5-6 mm) and deep (>6 mm) periodontal pockets in control, type 1 and type 2 diabetes groups of the study population

	Time	Group					
		CG		DM1		DM2	
		N	%	N	%	N	%
0-4 mm	Baseline	1474	84.62	1643	91.58	1314	88.13
	3 months	1614	92.76	1694	94.43	1360	92.90
5-6 mm	Baseline	203	11.65	139	7.75	152	10.19
	3 months	104	5.98	93	5.18	90	6.15
>6 mm	Baseline	65	3.73	12	0.67	25	1.68
	3 months	16	1.26	7	0.39	14	0.96

DM 1: type 1 diabetes mellitus; DM 2: type 2 diabetes mellitus; CG: control group.

**Fig 1:**
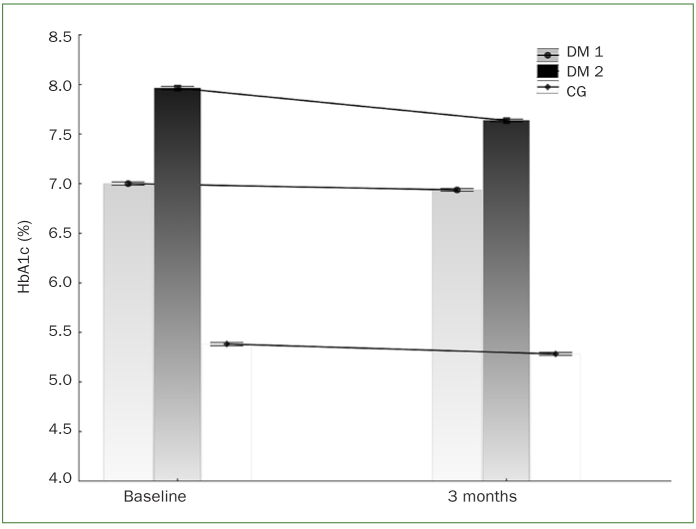
Glycated haemoglobin values (%) at baseline and three months after full-mouth disinfection. DM 1: type 1 diabetes mellitus; DM 2: type 2 diabetes mellitus; CG: control group.

HbA1c decreased in a statistically significant manner in all three groups (p < 0.05) three months after full-mouth disinfection was performed (Fig 1). The highest value measured in the DM 2 group dropped from a baseline value of 7.99 ± 1.02% to 7.64 ± 0.79% three months after FMD treatment. A lower baseline value of HbA1c in DM 1 group (7.01 ± 0.77%) decreased to a final value of 6.95 ± 0.82%. In the non-diabetic control group, HbA1c decreased slightly from baseline 5.38 ± 0.26% to 5.29 ± 0.21%.

### Microbiological Analysis

Table 4 shows the proportions of periodontopathogenic bacteria in periodontal pockets at baseline and 3 months after FMD for all three groups.

**Table 4:** Prevalence of periodontal bacteria (%) in type 1 and type 2 diabetes patients and in the control group at baseline and three months after full-mouth disinfection

	DM 1		DM 2		CG	
	Baseline	3 months	Baseline	3 months	Baseline	3 months
Aa (%)	18.1±4.0	40.9±5.0*	22.7±4.0	18.1±4.0	18.1±4.0	13.6±4.0
Pg (%)	27.2±4.6	27.3±4.6	72.7±4.6 §	68.2±4.8	68.1±5.0¶	59.1±5.0*
Pi (%)	41.0±5.0	41.0±5.0	45.5±5.1	50.0±5.1	59.1±5.0	40.9±5.0
Td (%)	81.8±3.9	73.0±4.6	82.0±3.9	77.2±4.3	91.0±2.9	81.8±3.9
Tf (%)	86.4±3.5	72.7±4.6	100±0	100±0	100±0	91.0±2.9

DM 1: type 1 diabetes mellitus; DM 2: type 2 diabetes mellitus; CG: control group. Aa: *Aggregatibacter actinomycetemcomitans*; Pg: *Porphyromonas gingivalis;* Pi: *Prevotella intermedia;* Tf: *Tannerella forsythia;* Td: *Treponema denticola*. FMD: full-mouth disinfection. *p < 0.05 vs baseline; § p < 0.05 vs baseline diabetes type 1; ¶ p < 0.05 vs baseline diabetes type 1.

The microbiological assessment showed that the proportion of all five periodontopathogenic bacteria (Aa, Pi, Pg Td and Tf) was reduced only in the control group 3 months after treatment. Only a decrease in Pg was statistically significant (p < 0.05).

In the type 1 diabetes group, the proportions of Tf and Td between baseline and 3 months after FMD decreased, while the proportion of Pg and Pi remained the same. The proportion of Aa increased significantly after FMD (p < 0.05).

In the type 2 diabetes group, the proportions of Aa, Pg and Td between baseline and 3 months after FMD decreased, the proportion of Tf did not change. The proportion of Pi increased, but the difference was not statistically significant.

The difference in the prevalence of Pg between type 1 and type 2 diabetes groups at baseline and type 1 diabetes and the control group at baseline was statistically significant (p < 0.05).

Tf was the most common bacteria found in samples of patients with type 1 diabetes mellitus, with a prevalence of 86.4%, followed by Td (81.8%), Pi (41.0%), Pg (27.2%) and Aa (18.1%).

Tf was also the most common bacterial species found in samples of patients with type 2 diabetes mellitus and in the control group, the prevalence being 100% in both groups, followed by Td (82.0% vs 91.0% respectively), Pg (72.7% vs 68.1%), Pi (45.5% vs 59.1%), and Aa (22.7% vs 18.1%) (Table 4).

## Discussion

The results of our study revealed that treatment with the full-mouth disinfection protocol improves the periodontal parameters and decreases the level of HbA1c in periodontal patients with type 1 and 2 diabetes, as well as in periodontal patients without diabetes. However, the proportion of all five periodontopathogenic bacteria decreased only in the control group (without diabetes). Our report supports the findings of some studies which also concluded that periodontal therapy improves glycaemic control in type 1 and 2 diabetes patients.^23,28^

HbA1c reflects the control of glycaemia in diabetic patients and can also be monitored chairside.^2^ Studies testing the precision of Point-of-Care HbA1c tests show that its analytical parameters are similar to methods performed in a laboratory.^4,27,34^ A study performed by Agarwal et al,^1^ who investigated the influence of periodontal treatment in type 2 diabetes patients with chronic periodontitis with the evaluation of HbA1c, also used a chairside method for the assessment of HbA1c, and recorded a statistically significant reduction in the clinical parameters and HbA1c. The use of chairside devices for the evaluation of glycaemic control of diabetes patients and also for detecting any increases in HbA1c in patients not yet diagnosed with diabetes would offer the dentist the chance to instantly detect any changes in HbA1c.

On the other hand, a study carried out by Dag et al^6^ did not show a statistically significant reduction in HbA1c levels after periodontal therapy. One possible explanation could be that the protocol of full-mouth disinfection more efficiently reduces periodontal parameters and glycated haemoglobin than classical scaling and root planing performed in more sessions, which was the method used in the Dag et al study.^6^

It has previously been considered that microbiota in diabetic patients are different compared to those without diabetes.^35^ New evidence suggests that microbial species found in patients with periodontal disease and without diabetes are the same, but the prevalence of certain species is higher in diseased sites.^15,22^

The microbiological assessment showed a reduction of bacterial load of all five periodontal pathogens after treatment only in the control group, and not in diabetes mellitus groups.

In our study, we found a statistically significant difference in the prevalence of Pg between type 1 and type 2 diabetes groups at baseline and type 1 diabetes and the control group at baseline. Thorstensson et al^31^ found significantly greater numbers of Pg in insulin-dependent diabetes patients compared to controls, which is in accordance with our study.

Tf and Td were the most frequently found bacteria in samples from type 1 and type 2 diabetes patients and also in the control group. This result is in accordance with a study carried out by Schara et al,^24^ which included type 1 diabetes patients and concluded that Tf and Td are the most frequently found species.

The proportion of Aa-positive sites in type 1 diabetes patients in our study increased, which is in accordance with the findings of a study by Petelin et al,^18^ where scaling and root planing in systemically healthy chronic periodontitis patients did not affect Aa but did affect the other 4 periodontal pathogens. Studies show that scaling and root planing effectively lowers the amount of Pg, Td, Tf bacteria of the red complex as defined by Socransky et al^26^ but has no effect on other bacteria.^5,10^ Aa is a rare bacterium of the mouth that penetrates into its soft tissues.^14^ For that reason, it is possible that Aa is not affected or can even increase by mechanical cleaning of the teeth. Furthermore, the gingival crevicular fluid of diabetes patients contains higher concentrations of glucose compared to patients without diabetes.^8^ Consequently, the food supply for subgingival microorganisms is altered and changes in proportions of bacteria can occur.^17^ That could explain the increase of Aa in type 1 diabetes patients in our study.

One of the limitations of our study was the low number of patients included in the study. Further investigations involving more patients are needed to obtain more accurate results. The changes should also be observed over a longer period of time. However, our study is one of the very few studies that investigated the influence of the FMD protocol on the level of glycated haemoglobin and on subgingival microbiota in patients with type 1 and type 2 diabetes.

## Conclusions

The full mouth-disinfection protocol improves periodontal parameters and glycaemic control of type 1 and type 2 diabetes patients with periodontitis.
